# Effect of nano silver and silver nitrate on seed yield of (*Ocimum basilicum* L.)

**DOI:** 10.1186/s13588-014-0011-0

**Published:** 2014-10-02

**Authors:** Fatemeh Nejatzadeh-Barandozi, Fariborz Darvishzadeh, Ali Aminkhani

**Affiliations:** 1 Department of Horticulture, Faculty of Agriculture, Khoy Branch, Islamic Azad University, Khoy, Iran; 2 Department of Horticulture, Faculty of Agriculture, Garmsar Branch, Islamic Azad University, Garmsar, P.O. Box 58168-44799, Iran; 3 Department of Chemistry, Khoy Branch, Islamic Azad University, Khoy, Iran

**Keywords:** Basil, Nano silver, Silver nitrate, Seed yield, Polyphenol compounds

## Abstract

**Background:**

The aim of this study was to evaluate the effect of nano silver and silver nitrate on yield of seed in basil plant. The study was carried out in a randomized block design with three replications.

**Results:**

Four levels of either silver nitrate (0, 100, 200 and 300 ppm) or nano silver (0, 20, 40, and 60 ppm) were sprayed on basil plant at seed growth stage. The results showed that there was no significant difference between 100 ppm of silver nitrate and 60 ppm concentration of nano silver on the shoot silver concentration. However, increasing the concentration of silver nitrate from 100 to 300 ppm caused a decrease in seed yield. In contrast, a raise in the concentration of nano silver from 20 to 60 ppm has led to an improvement in the seed yield. Additionally, the lowest amount of seed yield was found with control plants.

**Conclusions:**

Finally, with increasing level of silver nitrate, the polyphenol compound content was raised but the enhancing level of nano silver resulting in the reduction of these components. In conclusion, nano silver can be used instead of other compounds of silver.

## Background

Basil (*Ocimum basilicum* L.) is aromatic herbs that are used extensively to add a distinctive aroma and flavor to food. The leaves can be used fresh or dried as a spice. Essential oils extracted from fresh leaves and flowers can be used as aroma additives in food, pharmaceuticals, and cosmetics [1]-[3]. Traditionally, basil has been used as a medicinal plant in the treatment of headaches, coughs, diarrhea, constipation, warts, worms, and kidney malfunction [1]. Phytohormones and environmental stresses are the effective factors in controlling abscission process [4]. It is demonstrated that the ethylene has an important role in initiation of the abscission layer in different plants [5]. Ethylene activates the biosynthesis genes of hydrolytic enzymes, e.g., cellulose and polygalacturonase, which induces separation of plant organs from the main plant [6],[7]. In addition, the abscission process could be regulated by the other phytohormones such as auxin (IAA) and abscisic acid (ABA). The later induces the abscission process through stimulation of the ethylene biosynthesis while auxin is effective in delaying the abscission by reducing the sensitivity of cell to ethylene [4],[6],[8]. Abscission may be delayed by using some chemical components such as amino isobutyric acid and cobalt ions, (aminooxy) acetic acid, silver thiosulfate (STS), and silver nitrate (AgNO_3_) [8],[9]. Several studies demonstrated that spraying of silver ions decreases the flowers and flower bud abscission in orchid plant [10]. Additionally, it has been reported that silver ions decreased 100% flower abscission of *Alstroemeria* plant as compared to untreated flower within two first days [11]. Moreover, ethylene is involved on senescence of flowers in *Bougainvillea* plant while this process may be postponed by spraying silver thiosulfate [12].

Nano silver solution consisting silver ions in the size range of 10 to 100 nm, and it has more stability in comparison to other solutions. Nano silver particles, also, have more surface area in contact to outer space due to their small size. Thus, the amount of adhesion to the cell surface is increasing which lead to their higher efficacy [13]. Additionally, nano silver may affect the metabolism, respiration, and reproduction of microorganism [14]. For example, the effect of nano silver on extend maintenance period of leaves (from 2 to 21 days) in asparagus plant is reported. Also, during this period, the amount of ascorbate, chlorophyll, and fiber were more in treated leaves [15]. The effect of silver nitrate in delaying the abscission has been studied [15]; however, the influence of nano silver on seed abscission has not been reported. Therefore, this study was carried out to assess the possibility of using nano silver and silver nitrate in delaying the time of seed abscission in basil plant.

## Methods

This study was carried out in a completely randomized block design with three replicates in the field research of Islamic Azad University of Khoy (Iran). The data of this study was analyzed by SAS software, and the comparison was done according to least significant difference (LSD) test method. Four levels of either silver nitrate (0, 100, 200, and 300 ppm) or nano silver (0, 20, 40, and 60 ppm) were sprayed with 0.1% Tween 20 (Tween 20, Sigma-Aldrich, St. Louis, MO, USA) on basil plant at seed growth stage (that is 45 days after cultivation) and were repeated after 2 weeks. The nano silver solution with an average particle diameter of 25 nm was obtained from Pars Nano Nasb Company (Pars Nano Nasb Company, Tehran, Iran). Different parameters (that is, leave number, plant height, dry weight of plant, length and width of leaf, dry weight of inflorescence, seed yield, and weight of 100 seeds) were determined; greenness of leaves was measured by a chlorophyll meter. The biochemical properties included polyphenol and tannin were determined according to [16]. Concentration of silver in the plant shoot was measured by ‘inductively coupled plasma’ according to the method described by [16]. The principal operating parameters of the instrument were as follows: argon gas flow: auxiliary, 1 L/min, nebulizer (crossflow), 0.8 L/min; sample uptake: 60 s. Measurements were carried out in the axial mode at 328.068 nm.

## Results

**Figure 1:**
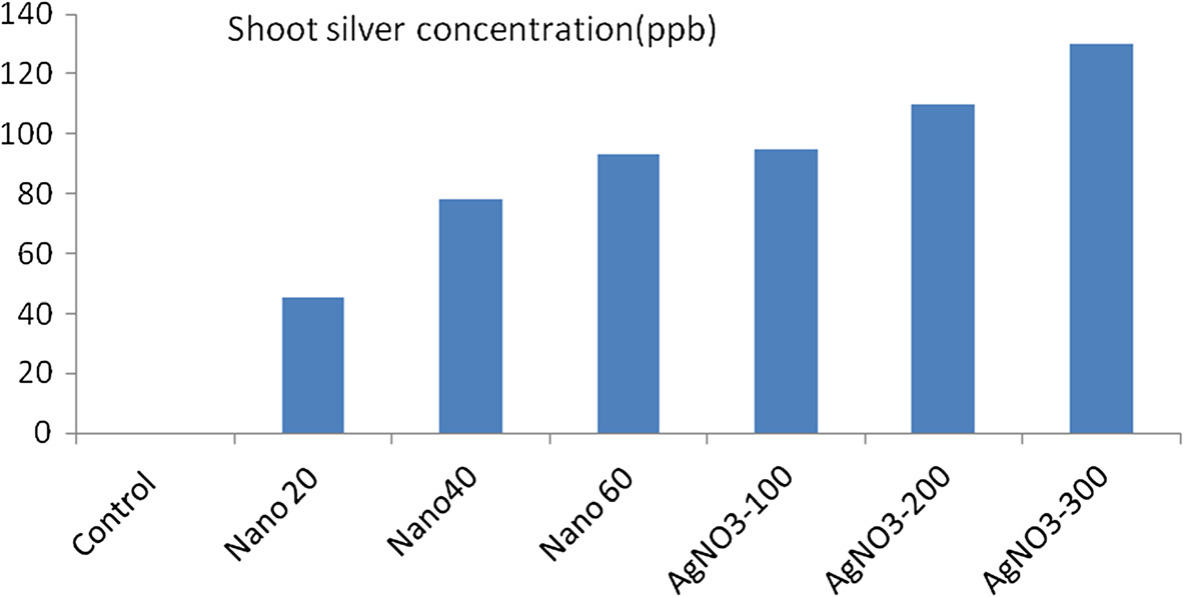
**Effect of nano silver and silver nitrate treatments on concentration of silver.** In plant tissue (μg/g dry tissue of plant).

Using silver (either as nano silver or silver nitrate) had a significant effect on silver concentration in the plant shoot, number of leaves, height of the plant, plant dry weight, inflorescence dry weight, seed yield, weight of 100 seeds, and polyphenol and tannin content in shoot (Table 1). In contrast, the length, width, and greenness of leaves have not affected by silver treatment (Table 1). Silver concentration in shoot was increased by all treatments (either nano silver or silver nitrate) However, the higher concentration of silver in the shoot was obtained by silver nitrate treatment in comparison to others (Figure 1). There was no significant difference between 100 ppm of silver nitrate and 60 ppm concentration of nano silver on the shoot silver concentration (Figure 1).

**Table 1:** Analysis of variance mean square testing traits

**SOV**	**Df**	**LN**	**PH**	**DWP**	**DWI**	**LL**	**LW**	**SY**	**WS**	**PP**	**TN**	**SC**
*R*	2	65.95 ns	34.01 ns	5446.50**	1401.62**	1.15 ns	1.85 ns	9.66**	0.060**	0.66*	0.13 ns	282.63*
*T*	6	315.84**	30.23*	3775.01**	794.02**	0.44 ns	0.23 ns	30.40**	0.044**	0.35*	0.26**	5888.46**
*E*	12	30.39	10.35	773.46	116.30	0.98	0.56	0.92	0.002	0.103	0.043	65.016
*CV*		10.20	8.64	9.40	5.70	10.14	14.22	4.63	3.40	11.78	11.7	9.06

* and ** respectively, significant levels of 5% and 1%. LN, leaf number; PH, plant height; DWP, dry weight of plant; DWI, dry weight of inflorescence; LL, leaf length; LW, leaf width; SY, seed yield; WS, weight of 100 seeds; PP, polyphenol; TN, tannin; SC, silver concentration.

The highest number of leaves was obtained by spraying 100 ppm of silver nitrate, while the lowest was seen in control plants (Table 2). The number of leaves and plant height was improved by increasing the concentration of nano silver while they were reduced by rising in silver nitrate concentration. The longest height of the plant was obtained with the treatment of nano silver 60 ppm, while the lowest amount of this feature was seen with control (Table 2). However, by going up, the concentration of silver nitrate from 100 to 300 ppm caused a decrease in the dry weight of plant and dry weight of inflorescence. In contrast, a raise in the concentration of nano silver from 20 to 60 ppm has led to an improvement in those parameters (Table 2). Raising the concentration of silver nitrate from 100 to 300 ppm caused a decline in the seed yield, but an increase in concentration of nano silver from 20 to 60 ppm leads to arise in the seed yield. The lowest amount of seed yield was found with control samples, and the highest was obtained by silver nitrate 100 ppm treatment. However, seed yield was found to be more than the control sample with different concentrations of nano silver and silver nitrate (Table 2). The highest weight of 100 seeds was observed in the control sample, while this weight was decreased when either nano silver or silver nitrate was applied (Table 2).

**Table 2:** Comparison mean effect of different concentrations of nano silver and silver nitrate on test traits

**Treatment**	**LN**	**PH (cm)**	**DWP (g/m**^**2**^**)**	**DWI (g/m**^**2**^**)**	**LL (cm)**	**LW (cm)**	**SY (g/m**^**2**^**)**	**WS (g)**	**PP**	**TN**
Control	41.530e	30.977c	246.94d	161.90d	8.300a	5.040a	14.62e	2.01e	3.160a	2.220a
NS 20 ppm	46.24de	34.68abc	268.38 dc	174.68 cd	8.324a	5.367a	18.10d	1.860b	2.941ab	1.985ab
NS 40 ppm	50.06cde	38.392ab	301.50abc	188.10bc	8.501a	5.351a	20.41c	1.761bc	2.781abc	1.779bc
NS 60 ppm	53.364 cd	41.445a	347.06a	203.1ab	8.910a	5.701a	22.68b	1.70 cd	2.420ab	1.605 dc
NS 100 ppm	68.130a	39.810ab	325.79ab	210.45a	9.321a	5.725a	24.90a	1.615d	2.208c	1.265d
NS 200 ppm	67.431ab	7.025abc	303.72abc	199.8ab	9.090a	5.110a	23.14b	1.714 cd	2.595abc	1.672bc
NS 300 ppm	57.465bc	33.500bc	278.65bcd	189.30bc	9.920a	5.028a	19.71 cd	1.785bc	3.048a	1.960abc
LSD	9.965	5.750	49.660	19.268	1.765	1.350	1.725	0.105	0.570	0.376

Mean values, followed by the same letters in each column are not significantly different (Duncan's multiple range test at 5%). NS, nano silver; SN, silver nitrate; LN, leaf number; LN, leaf number; PH, plant height; DWP, dry weight of plant; DWI, dry weight of inflorescence; LL, leaf length; LW, leaf width; SY, seed yield; WS, weight of 100 seeds; PP, polyphenol, TN, tannin.

The highest content of polyphenol and tannin was observed in control, and the lowest content was observed with silver nitrate 100 ppm (Table 2). The polyphenol and tannin content was raised with the increasing level of silver nitrate but was decreased with the increase in nano silver. The greenness, length, and width of the leaves were not affected by nano silver and silver nitrate treatments (Table 1).

## Discussion

**Figure 2:**
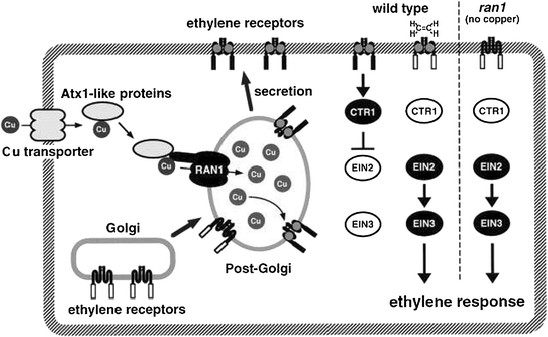
**A model for the function of RAN1 in the ethylene signaling pathway.** RAN1 is presumed to be localized in the membrane of a post-Golgi compartment. Copper ions received from CCH, a putative copper chaperon, is transported by RAN1 into a post-Golgi compartment, delivering the metal to membrane-targeted ethylene receptor apoproteins that become able to coordinate ethylene after the incorporation of copper ions. In the absence of the hormone, the receptors are active and negatively regulate downstream signaling components, preventing hormone-response phenotypes. Ethylene is expected to inactivate the receptors upon binding, presumably by causing a reduction in histidine kinase/phosphatase activity. This, in turn, results in derepression of downstream signaling components (EIN2, EIN3) and activation of hormone-response phenotypes. The metal-deficient ethylene receptors are nonfunctional, resulting in a constitutively activated signaling pathway.

**Figure 3:**
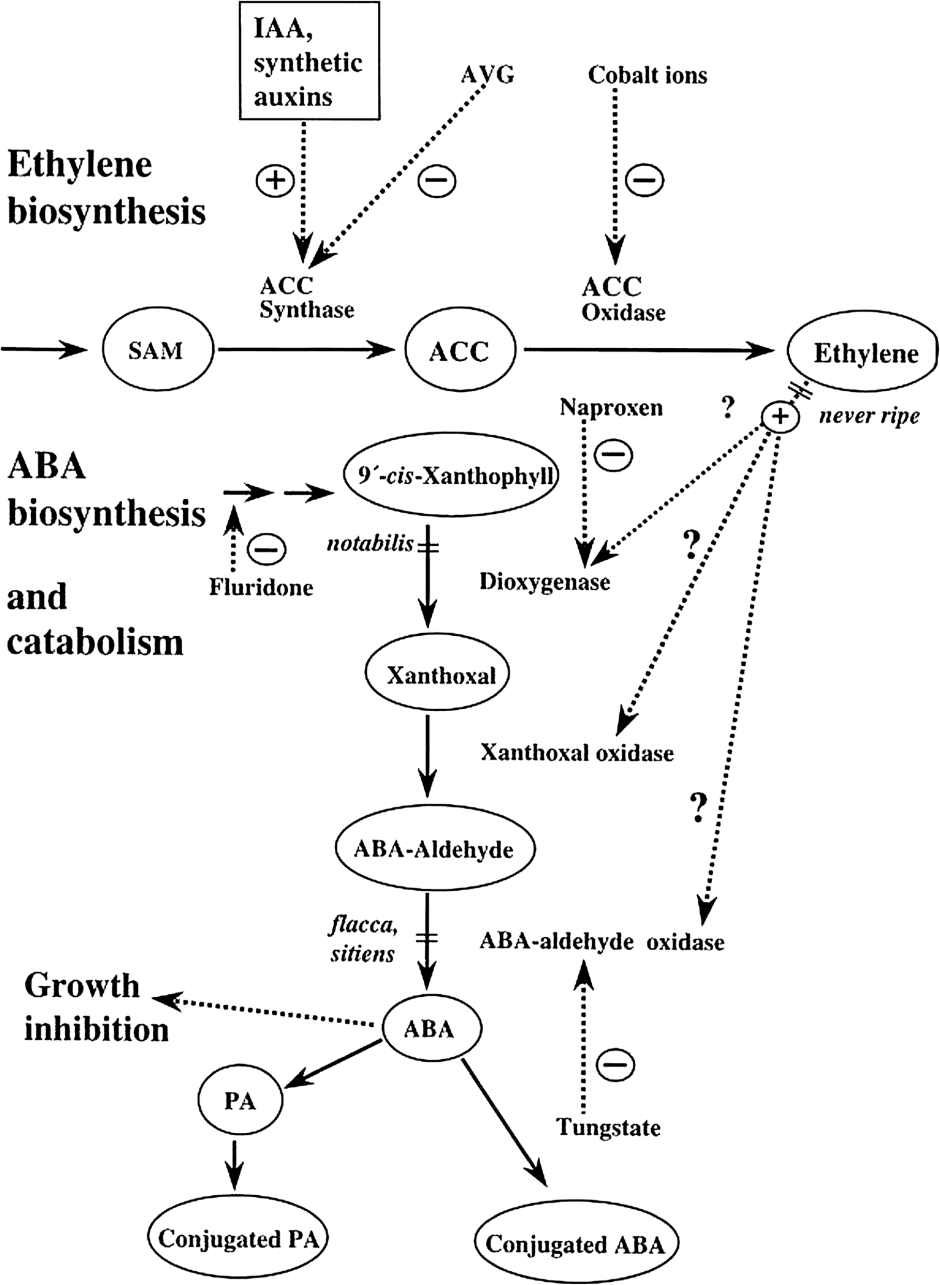
**Inhibit biosynthesis of ethylene.** Target sites of inhibitors (dotted arrows) are indicated. SAM, *S*-adenosyl-methionine.

Application of silver ions can displace copper ions from the receptor proteins (Figure 2) consequently, block ethylene perception, since copper ions have a critical role in ethylene binding upon receptors [17]-[19]. This effect of silver ion on ethylene was reported by several researchers [11],[20],[21]. Therefore, some results in this study can be referred to the effect of silver in preventing the ethylene action (Figure 3). Decrease of plant's height due to ethylene is proven. For example, mutants of tobacco and *Arabidopsis*, which synthesize a high concentration of ethylene, have lesser height as compared to their wild species [22]. Furthermore, Nichols and Kofranek [23] reported that silver ion causes the increase in stem height of tulips and rose plants. Under the *in vitro* conditions, silver nitrate inhibited biosynthesis of ethylene and caused regeneration of multiple shoots from hypocotyl sections of cotton [24]. Another researcher reported that silver nitrate is effective in increasing wet weight of tobacco [25]. Ethylene caused an increase in enzyme activity of chlorophyllase and destruction of internal membrane of chloroplast, while that 100 ppm of silver nitrate caused decreasing the production of ethylene and destruction of chlorophyll in calamondin fruit [26]. Spraying silver nitrate causes improvement of seed yield in wheat [15]. In the present experiment, foliar application of either nano silver or silver nitrate causes increase in seed yield as compared to control (Table 2). In basil plant, rise in seed yield can be due to increase in the number of inflorescence in unit area, number of seed in the inflorescence, weight of seeds, and decrease in seed abscission. Considering that the seed yield has a significant positive correlation with dry weight of inflorescence (*r* = 0.808, *p* < 0.0001), but it has a significant negative correlation with weight of 100 seeds (*r* = −0.835, *p* < 0.0001). Consequently, it can be concluded that the increase of seed yield in this study is due to the increase of the inflorescence number in unit area and decrease in seed abscission.

Seed abscission is one of the main factors in reducing seed yield in basil plant. It is proven that one of the reasons for plant organ abscission is imbalance between phytohormones. Ethylene is playing an important role in this process. Furthermore, it is proven that silver ions inhibit the ethylene action by preventing its connection to its receptors in plant cells [ 8, 6, 4]. Thus, the increase in the seed yield was a result of reducing the seed abscission due to the inhibitory effect of silver on ethylene action. These results are confirmed by the results obtained from other studies [10],[11],[15],[20]. There are many reports about the use of silver nitrate in decreasing the abscission, but this study is the first research which is about nano silver effect on decreasing the abscission of reproductive organs of plants. Increasing silver concentration in aerial organs of sprayed plants with nano silver caused decreasing polyphenol and tannin content. While there was an increased in polyphenol and tannin content by spraying silver nitrate due to rising of the silver concentration in aerial organs of plant. There are many reports available about ethylene effect on increasing the phenol content [27],[28]. On the other hand, high concentration of heavy metals (that is silver) in plant tissue causes polymerization of phenol by peroxidase enzyme which chelates the heavy metals [28],[29]. Therefore, by increasing the level of silver nitrate more than 100 ppm, phenol and tannin content enhances due to toxicity effect of silver on plant cells.

## Conclusions

There was no significant difference between 100 ppm of silver nitrate and 60 ppm concentration of nano silver on the shoot silver concentration. Therefore, permeability of nano silver is far greater than that of silver nitrate. The reason of this matter is the small size of nano particle, which causes more adhesion of nano particles to plant tissues. By considering the lesser use of silver in nano silver, this treatment can be used instead of other combinations of silver. However, the nano silver effect compared with other silver combinations on reducing the ethylene effect needs more researches.

## Competing interests

The authors declare that they have no competing interests.
